# Gamma oscillatory activity in vitro: a model system to assess pathophysiological mechanisms of comorbidity between autism and epilepsy

**DOI:** 10.1038/s41398-017-0065-7

**Published:** 2018-01-10

**Authors:** D. Subramanian, E. Pralong, R. T. Daniel, A. G. Chacko, R. Stoop, K. S. Babu

**Affiliations:** 1 Department of Neurological Sciences, Christian Medical College, Vellore, India; 2 Department of Clinical Neurosciences, Lausanne University Hospital, Lausanne, Switzerland; 3 Department of Psychiatry, Center for Psychiatric Neuroscience, Lausanne University Hospital, Lausanne, Switzerland

## Abstract

Autism spectrum disorder (ASD) and temporal lobe epilepsy exhibit remarkable comorbidity, but for reasons not clearly understood. To reveal a common pathophysiological mechanism, we here describe and characterize an in vitro epileptiform activity in the rat hippocampus that exhibits common features with in vivo activity in rodent ASD models. We discovered the development of this activity in the CA1 region of horizontal slices after prolonged interictal-like epileptiform activity in the CA3 region that was provoked by incubation in high potassium artificial cerebrospinal fluid. The CA1 epileptiform bursts were insensitive to blockers of glutamatergic transmission, and were carried by synaptic as well as extrasynaptic, tonically activated gamma-aminobutyric acid type A (GABA(A)) receptors. The bursts bear resemblance to in vivo gamma-oscillatory activity found in rat ASD models with respect to their gamma frequency spectrum, their origin (in the CA1), and their sensitivity to blockers of cation-chloride pumps (NKCC1 and KCC2), as well as to oxytocin. Considering this bursting activity as an in vitro model for studying comorbidity between epilepsy and ASD may help to disentangle the intricate interactions that underlie the comorbidity between both diseases and suggests that extrasynaptic tonic GABAergic transmission could represent a potential target for ASD.

## Introduction

Epilepsy and autism spectrum disorder (ASD) are developmental disorders that exhibit a high degree of comorbidity and for which, despite decades of research, effective treatments are still lacking^1–3^. The increased prevalence of clinical epilepsy and paroxysmal electroencephalographic (EEG) epileptiform activity in patients with ASD has led to the belief that ASD and epilepsy might have a partially common underlying brain pathology^2^. Although their comorbidity could be caused at the level of the chromosome or by co-occurring environmental influences, recent findings rather suggest a common pathophysiological mechanism at the level of neuronal circuits as underlying cause^1^.

One possible common mechanism underlying ASD and epilepsy may be found in an early excitation/inhibition imbalance in the brain^4–6^. ASD has been postulated to be caused by a reversal of gamma-aminobutyric acid type A (GABA(A)) receptor-mediated neurotransmission from inhibition to excitation as a result of an increased intracellular Cl^−^ concentration ([Cl^−^]_i_) that would result in reduced inhibition in the ASD brain^5,7,8^. Such disruptions in Cl^−^ homeostasis have indeed been reported in both humans and animal models of autism^7,9–11^. Consequently, drug interventions targeting the cation-chloride co-transporters within the CA1/CA3 region of the hippocampus have been able to rescue ASD-associated changes in intracranially recorded gamma oscillations as well as behavioral expressions such as ultrasonic vocalizations^5,12^. Among these are the diuretic bumetanide and, more recently, oxytocin, a naturally occurring neuropeptide that shows promise for the treatment of autism^9,10,12^.

We therefore based our present approach on the assumption that the disruption of the chloride equilibrium potential can also underlie epileptic pathologies^13,14^ and can thus constitute a basis for a common pathophysiological mechanism. To test this hypothesis, we developed a rodent in vitro model of epileptiform activity which we characterized in close comparison with established in vivo recordings from rodent ASD models and on which we compared effects of pharmacological interventions already employed in ASD. We found a bursting activity in the CA1 hippocampal region that was primarily driven by GABA(A) receptor activation and that exhibited strong 40 Hz components which closely resembled atypical gamma oscillations observed in in vivo animal models of ASD. We validated our model by testing pharmacological treatments that are efficient in animal models of ASD. Our findings revealed a new anti-convulsant potential for oxytocin and, in addition, we found that extrasynaptic GABAergic transmission appears to underlie these epileptiform bursts which may thus point to new potential targets for epilepsy and ASD.

## Materials and methods

### Animals

All procedures were conducted in accordance with the guidelines of the Committee for the Purpose of Control and Supervision of Experiments on Animals and were approved by the Institutional Review Board and Institutional Animal Ethics Committee of Christian Medical College, Vellore.

### Slice preparation

After anesthesia with isoflurane and decapitation, brains from 4 to 8 weeks Wistar rats were removed quickly and placed in ice-cold cutting solution containing (in mM): 110 sucrose, 60 NaCl, 3 KCl, 0.5 CaCl_2_, 7 MgSo_4_, 26 NaHCO_3_, 1.2 NaH_2_PO_4_, 5 glucose at pH 7.4, and oxygenated with 95% O_2_/5% CO_2_. Horizontal slices of the ventral hippocampus (450 µm thick, see Stoop and Pralong^15^) were cut using a vibratome (VF200, Precisionary instruments, USA) and perfused in an interface chamber for 1 h or more at 24–26 °C in a solution of artificial cerebrospinal fluid (aCSF) containing (in mM) 124 NaCl, 3.5 KCl, 2 CaCl_2_, 2 MgSO_4_, 26 NaHCO_3_, 1.2 NaH_2_PO_4_, and 10 glucose at a rate of 1 ml/min. A fine needle (31 G) was used for dissecting out entorhinal cortex (EC) before induction of epileptiform events and for disconnecting Schaffer collateral (SC) projections from CA3 to CA1, or for preparing CA1 mini-slices by isolating CA1 between CA3 and the subiculum.

### Electrophysiological recordings and induction of epileptiform events

Field potentials were recorded from the stratum pyramidale of CA3a and CA1 subfields using aCSF filled microelectrodes (~ 1MΩ). Recorded signals were amplified 10,000 times, band pass filtered between 1 and 1000 Hz (Model 1800, AM Systems, USA), and digitized at 5 kHz (PCle 6321-DAQ-National Instruments, USA), using custom-written codes for LabView (Ver-10.0, National Instruments). Ten minutes of baseline were recorded before any intervention and data were further filtered between 1 and 100 Hz during analysis. Incubation in aCSF with 8.5 mM KCl and 1.2 mM CaCl_2_ and MgSO_4_ at 34–36 °C (TC-344B Dual automatic temperature controllers, Warner Instruments, USA) produced epileptiform events that were identified and classified as (i) interictal-like epileptiform discharges (hereafter referred as interictal discharges) and (ii) ictal-like epileptiform discharges (hereafter referred as IEDs) based on their duration and appearance as described by Dzhala and Staley^16^.

### Statistics and analysis

Sample size was determined prior to experiments to achieve a power of 0.80 and a probability of type I error (*α*) of 0.05. Parameters studied included number of spikes/event, peak amplitude, burst duration, spike frequency, interburst interval, and power of gamma oscillations. Slices showing spreading depression were excluded from the study since they can affect the parameters evaluated. For event detection, burst, and spectral analysis we used Clampfit (Molecular Devices, USA). Bursts were grouped together based on their inter-spike intervals ( < 500 ms). Results are presented as mean or normalized percentage difference ± standard error of mean (SEM). For statistical analysis, paired and unpaired two-tailed Student's *t* test was used. Significance was assigned at *p* < 0.05.

### Chemicals

Bicuculline methiodide (BMI), picrotoxin, SKF 89976A, and aCSF ingredients originated from Sigma-Aldrich (St. Louis, MO, USA); 2,3-dioxo-6-nitro-1,2,3,4- tetrahydrobenzo[*f*]quinoxaline-7-sulfonamide disodium salt (NBQX), D-AP5, bumetanide, CLP257, and VU 0463271 from Tocris Bioscience (Bristol, UK); [Thr4, Gly7] oxytocin (TGOT) and (d(CH2)51,Tyr(Me)2,Thr4,Orn8,des-Gly-NH29)-vasotocin (vasotocin) from American Peptide Company (Sunnyvale, CA, USA).

## Results

**Fig. 1:**
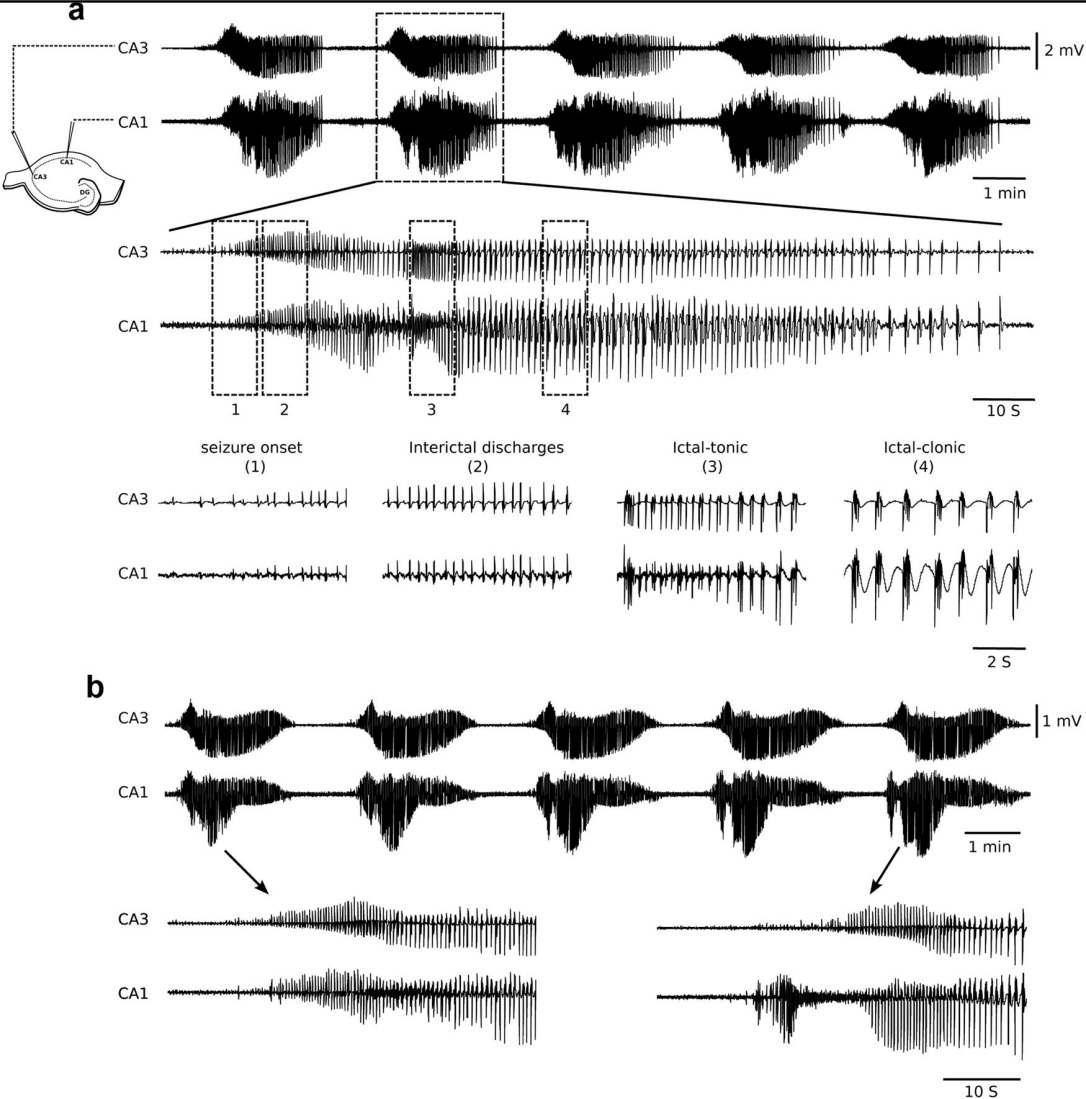
High K^+^ aCSF induced epileptiform discharges in hippocampal slices. **a** Trains of epileptiform discharges are seen in CA3 and CA1 within 40–60 min of incubation (*n* = 34 slices from 27 animals). A single epileptiform event and its different phases are shown expanded in time^1–4^. **b** Prolonged incubation in high K^+^ aCSF leads to the development of CA1 gamma bursts (*n* = 60 slices from 44 animals) subsets below show the initiation of epileptiform event before (left) and after the development of gamma bursts. Note the gamma bursts appearing in CA1 before initiation of epileptiform activity in CA3

We found that within 40–60 min of exposure to aCSF with high K^+^, “seizure onset” in both CA3 and CA1 (Fig. 1a1) was followed by typical interictal-like discharges (Fig. 1a2, 0.67 ± 0.08 mV amplitude, 22.8 ± 2.4 s intervals) that progressed into IEDs. These latter consisted of an initial period of sustained discharges (“ictal tonic”, Fig. 1a3, 1.1 ± 0.15 mV, 8.6 ± 0.8 s), followed by intermittent discharges (“ictal clonic”, Fig 1a4, 1.5 ± 0.2 mV, 66.3 ± 8.6 s). Complete IEDs consisted of 361 ± 10.3 discharges that lasted 112 ± 2.4 s and occurred at intervals of 186 ± 3.8 s. They seemed to progress from CA3 with 15.2 ± 1.4 s delay between seizure onset to CA1 (Fig. 1a1).

**Fig. 2:**
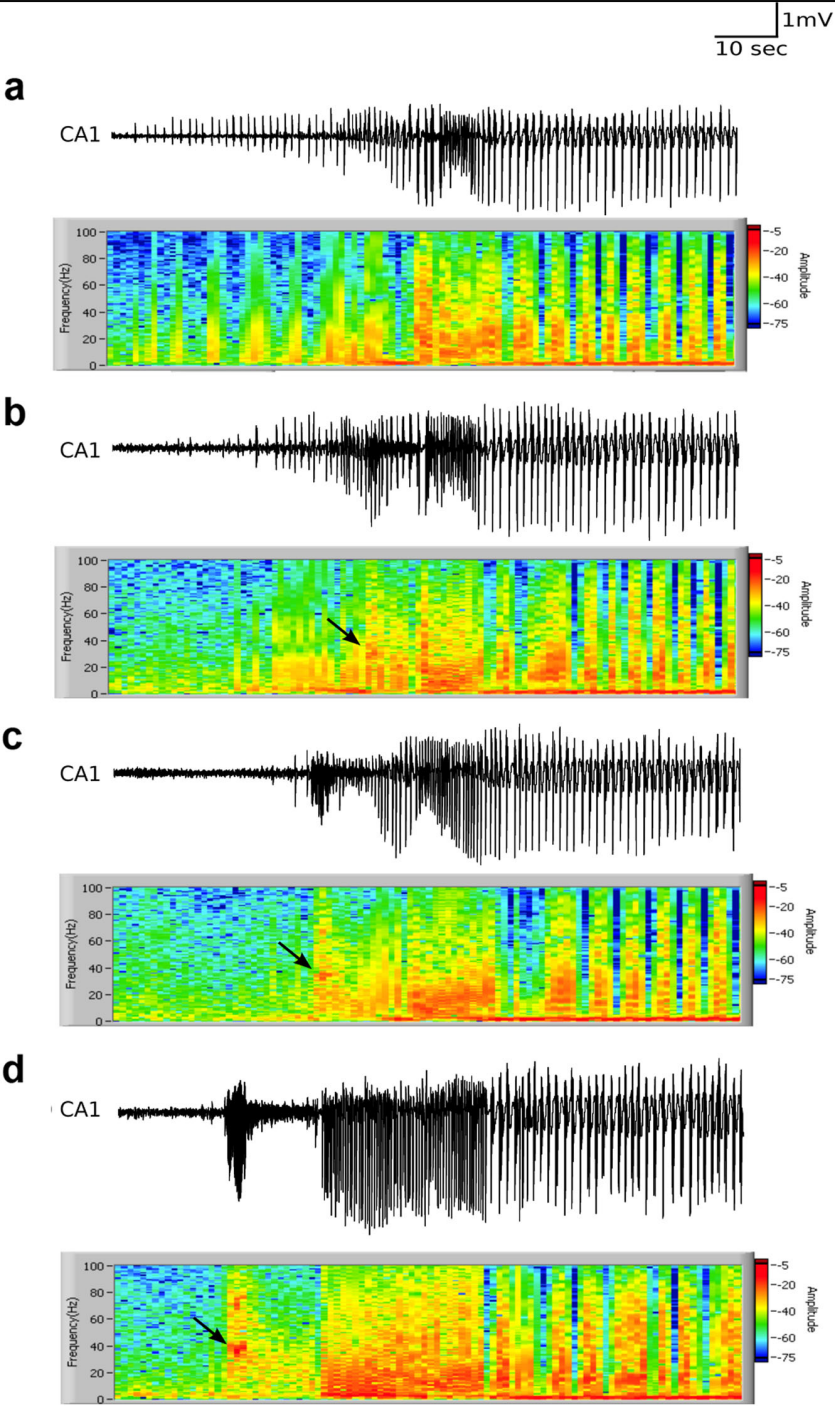
Spectrogram of CA1 field recordings during the development of CA1 gamma bursts. **a–d** The transition in initiation of epileptiform events in CA1 of the same slice at intervals of 5 min. The arrows denote the peak activation during gamma bursts which was mostly seen around 30–40 Hz

Upon prolonged incubation in high K^+^ aCSF (>60 min), we found a new type of bursts in the CA1 that preceded the IEDs. These CA1 bursts were of high frequency, short duration (4.2 ± 0.8 s), and consisted of 75 ± 13 population spike discharges with a peak amplitude of 1.3 ± 0.2 mV (*n* = 60 slices, 44 animals). They developed exclusively in the CA1 region and not only preceded interictal discharges and IED’s in the CA1 (by 8.9 ± 1 s), but also those in the CA3 region (Fig. 1b, right lower panel). To investigate if these CA1 bursts affected initiation of epileptiform events in CA3, we recorded field potentials simultaneously from CA3, CA2, and CA1. Although an increase in baseline activity was evident in CA2 (delay of 2 ± 0.3 s, Fig. 4a) and CA3 (delay of 8.6 ± 1.3 s), we could not conclusively determine such influence since properties of CA3 discharges appeared unaltered after the CA1 bursts developed (data not shown). Detailed spectral analysis of the CA1 bursts revealed a strong gamma frequency component (34.9 ± 1.7 Hz, Fig. 2), and we henceforth refer to these as “gamma bursts”.

**Fig. 3:**
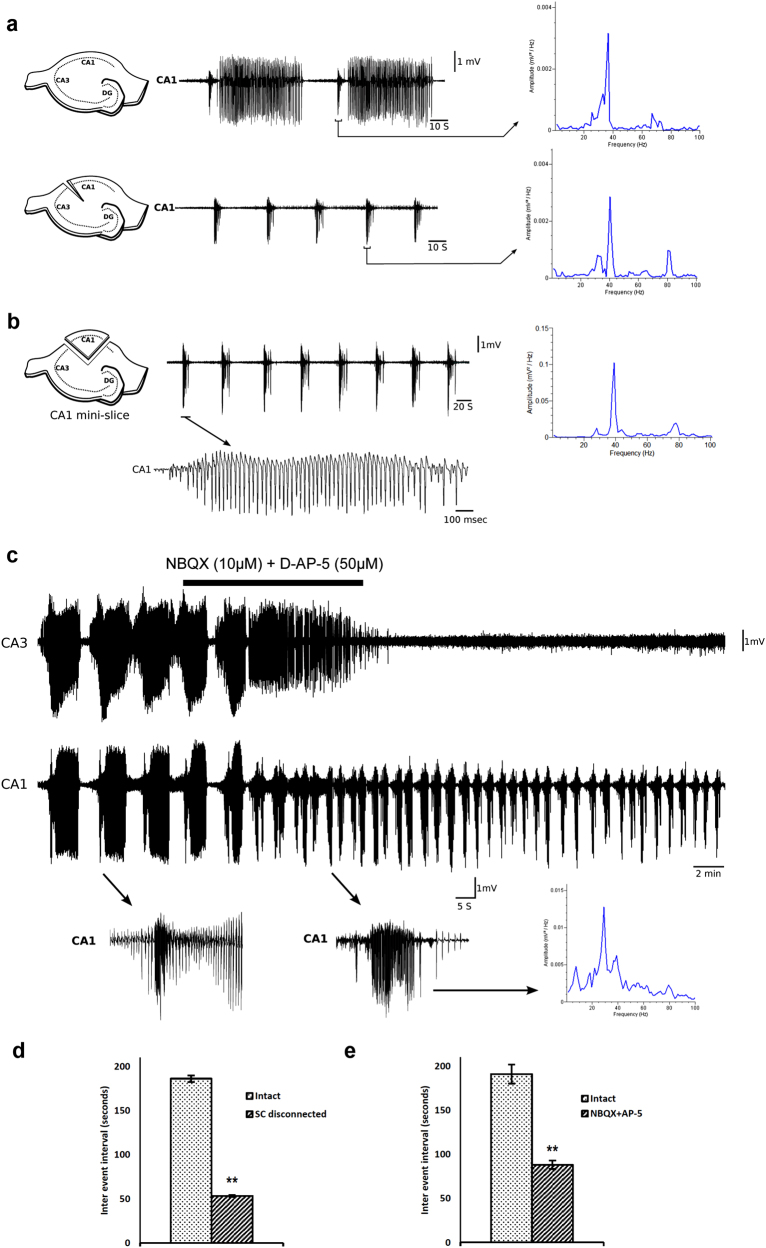
Anatomical and pharmacological isolation of CA1 from CA3 inputs allows for more frequent gamma bursts in CA1. **a** CA1 gamma bursts before and after disconnection of SC pathway with their corresponding power spectrum. Note that gamma bursts start appearing more frequently after SC disconnection (*n* = 14 slices from 10 animals). **b** Gamma bursts generated in isolated CA1 mini-slices with a single gamma burst expanded below with its corresponding power spectrum (*n* = 41 slices from 28 animals). **c** Pharmacological isolation of CA1 from CA3 inputs by NBQX (10 µM) and D-AP5 (50 µM). Note that all activity in CA3 is abolished by NBQX+D-AP5 and only gamma bursts remain in CA1 (*n* = 8 slices from eight animals). Subsets below show a gamma burst in CA1 before (left) and in the presence of NBQX+D-AP5. **d** Similar to anatomical disconnection of CA3 inputs; **e** pharmacological isolation of CA1 also causes decrease in intervals between gamma bursts. Averages ± standard error of the mean. **p = < 0.01 significance, unpaired Student's *t* test

**Fig. 4:**
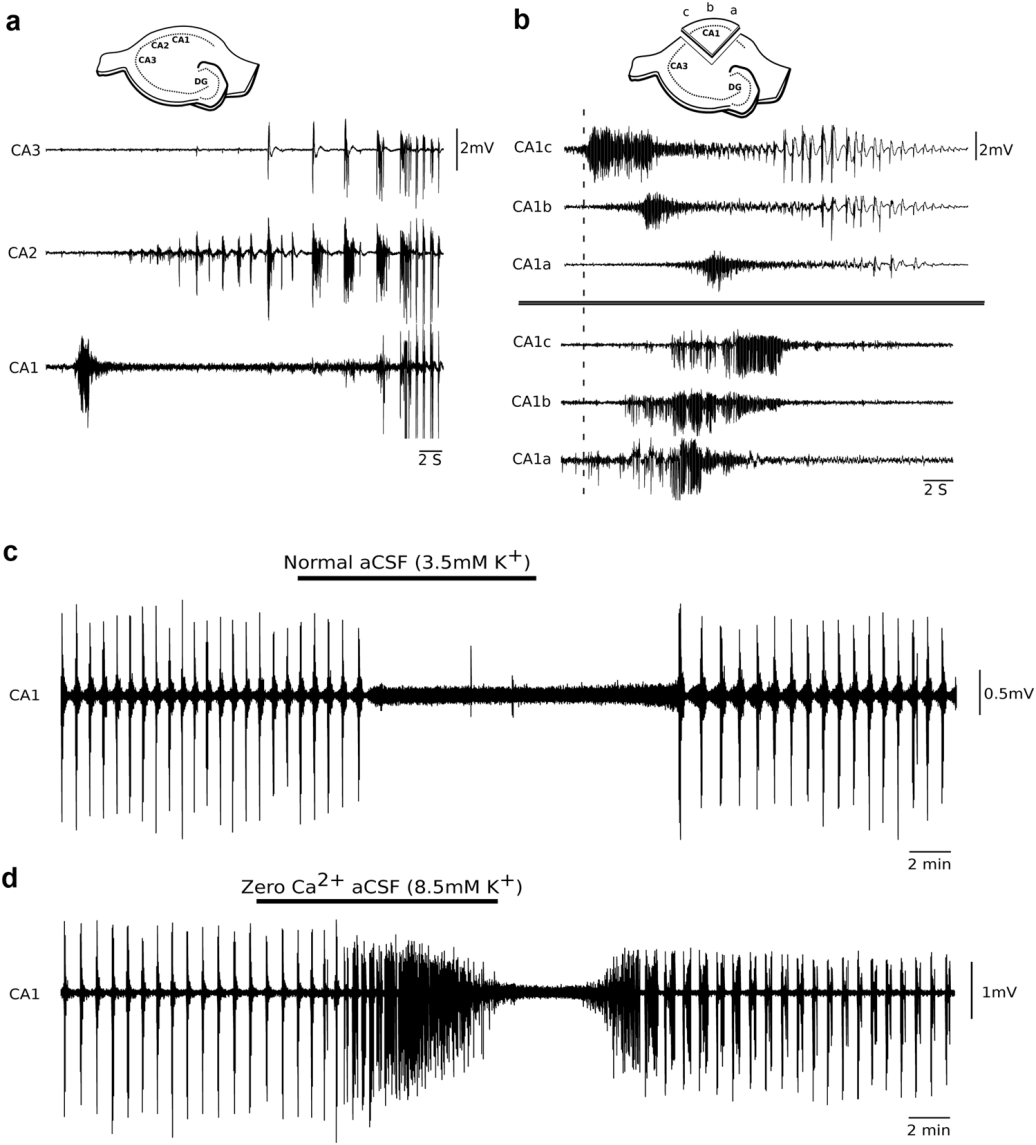
Propagation of CA1 gamma bursts. **a** Simultaneous field recordings from CA3, CA2, and CA1 show gamma bursts are confined to the CA1 where they begin before initiation of CA3 epileptiform events. **b** Simultaneous field recordings from CA1a, CA1b, and CA1c reveal that gamma bursts in most cases develop in CA1c from where they propagate towards CA1b and CA1a (*n* = 14 slices from nine animals). **c** Normal aCSF reversibly stops the generation of gamma bursts in CA1 mini-slices (*n* = 5 slices from five animals). **d** Perfusion of zero Ca^2+^ aCSF also reversibly blocks gamma bursts in CA1 mini-slices (*n* = 7 slices from five animals)

To examine the origin of these gamma bursts, we severed SC projections to the CA1 with the tip of a fine 31 G needle. Although this completely abolished IED’s in the CA1 region, it did not affect the gamma bursts (Fig. 3a). In fact, gamma bursts now appeared more often at shorter intervals (53.2 ± 1.3 s compared to 186 ± 3.8 s, Fig. 3a, d; *p* < 0.001, *n* = 14 slices, 10 animals). To further investigate if CA3 inputs are required for the initial development of gamma bursts, we prepared, before incubation in high K^+^ aCSF, isolated slices of the CA1, and henceforth referred to as “CA1 mini-slices” (Fig. 3b). In these CA1 mini-slices, gamma bursts appeared within 30 min of incubation without prior development of IEDs. Although in some mini-slices gamma bursts were followed by a series of after-discharges (see example Fig. 4b, top traces), in all other respects these gamma bursts exhibited similar characteristics as gamma bursts that had developed in intact slices in which SCs were severed (see Table 1 for spikes/burst, spike frequencies, peak amplitudes, burst intervals, and duration).

**Table 1:** Properties of gamma bursts in intact hippocampal slices and CA1 mini-slices

	Spikes/burst (number)	Spike frequency (Hz)	Peak amplitude (mV)	Burst duration (s)	Interburst interval (s)	*n*
Intact slices	75 ± 13	34.9 ± 1.7	1.3 ± 0.2	4.2 ± 0.8	189.4 ± 15.8	60 slices from 44 animals
NBQX+D-AP5 in intact slices	127.7 ± 6.7**	31.6 ± 1	2.2 ± 0.3**	8 ± 0.6**	88 ± 4.9**	8 slices from 8 animals
Schaeffer collateral disconnected	78.1 ± 2.3	35.4 ± 0.7	1.7 ± 0.2	3.9 ± 0.2	53.2 ± 1.3**	14 slices from 10 animals
CA1 mini-slice	86.4 ± 1.6	33.1 ± 0.4	1.8 ± 0.9	4.2 ± 1.6	56.2 ± 0.9**	6 slices from 6 animals

Table also shows the effect of glutamate receptor antagonist NBQX and D-AP5 in intact slices and CA1 mini-slices. Mean ± standard error of the mean; **p = < 0.01 significance compared to intact slices, unpaired Student's *t* test.

*D-AP5* D-2-amino-5-phosphonopentanoate, *NBQX* 2,3-dioxo-6-nitro-1,2,3,4- tetrahydrobenzo[*f*]quinoxaline-7-sulfonamide disodium salt

**Fig. 5:**
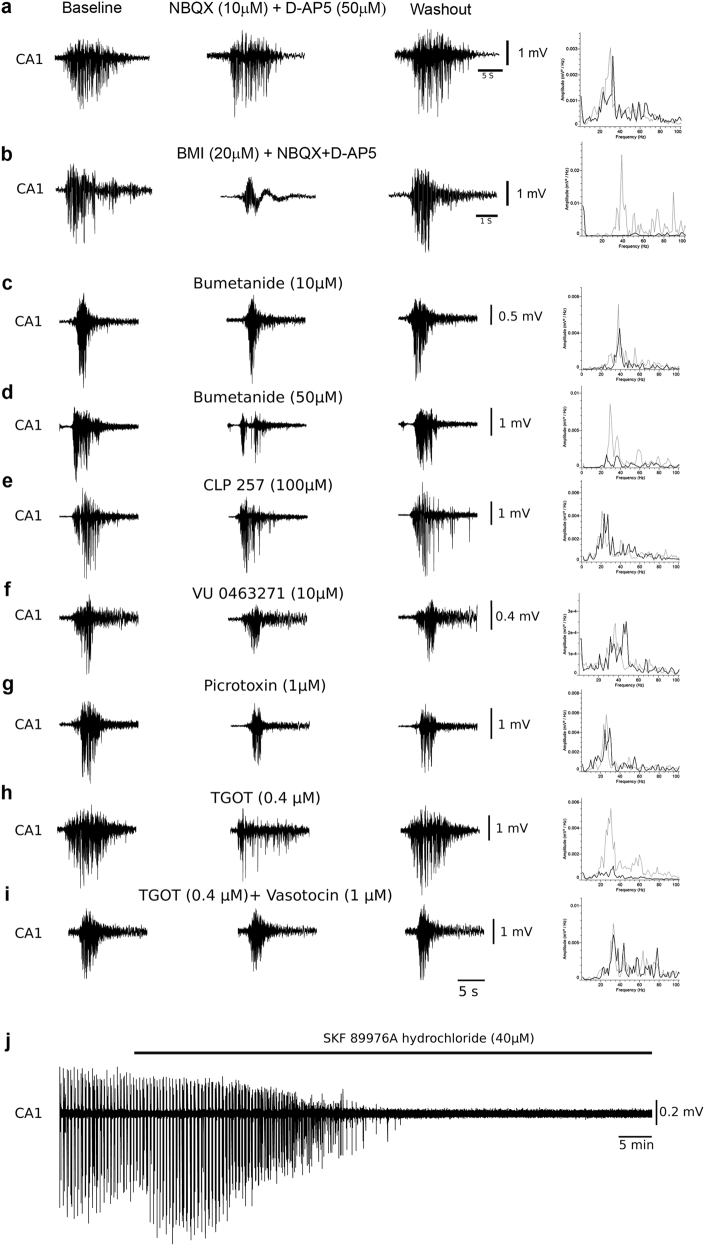
Characterization of synaptic transmission underlying gamma bursts in CA1 mini-slices. **a** NBQX (10 µM) and D-AP5 (50 µM) have a mild effect on the gamma bursts (*n* = 6 slices from 6 animals). **b** BMI (20 µM) significantly reduced the gamma bursts and completely abolished gamma frequency activity (*n* = 8 slices from 6 animals). **c** Application of NKCC1 blocker bumetanide (10 µM) significantly reduced gamma burst in CA1 mini-slices (*n* = 8 slices from 6 animals). **d**, **f** Higher concentration of bumetanide (50 µM; *n* = 11 slices from 8 animals) known to block KCC2 and specific KCC2 blocker VU 0463271 (10 µM; *n* = 7 slices from 5 slices) significantly affect gamma bursts. **e** Selective KCC2 activator CLP257 (100 µM) increases the interburst interval and reduces the peak amplitude, without significantly affecting other parameters (*n* = 6 slices from 6 animals, see Table 2 also). **g** Picrotoxin (1 µM) known to specifically block extrasynaptic GABA(A) receptors strongly attenuates the gamma bursts (*n* = 6 slices from 5 animals). **h** Oxytocin receptor agonist TGOT (0.4 µM) significantly reduces gamma bursts (*n* = 15 slices 9 animals). **i** The effect of TGOT is reduced in the presence of specific oxytocin receptor antagonist vasotocin (1 µM) (*n* = 6 slices from 5 animals). **j** Specific GAT-1 blocker SKF89976A hydrochloride (40 µM) completely abolishes gamma bursts in CA1 mini-slices (*n* = 8 slices from 6 animals). In spectrograms, gray trace represents before drug treatment and black traces represent in the presence of drug

To further examine their pharmacological sensitivity, we blocked glutamate transmission in intact slices with NBQX (10 μM) and D-AP5 (50 μM). Although this completely abolished IEDs in CA3 and CA1, it did not block the generation of CA1 gamma bursts. Instead, similar to anatomical disconnection of SC, it significantly shortened the intervals between consecutive gamma bursts (from 189.4 ± 15.8 to 88 ± 4.9 s, Fig. 3c, e). Furthermore, NBQX + AP5 also significantly increased the average spikes/burst, burst duration, and amplitude (see Table 1). Similar effects of NBQX + D-AP5 were found in CA1 mini-slices (Fig. 5a, Table 1). In summary, these experiments show that gamma bursts do not depend on glutamatergic synaptic transmission, and that they are generated and can develop in the CA1 independently from CA3 inputs.

To identify the precise origin of the gamma bursts within the CA1 subfield, we simultaneously recorded field potentials in CA1a, CA1b, and CA1c in mini-slices. In most slices (9/14), gamma bursts originated from CA1c and propagated toward CA1b and CA1a (with delays of 1.7 ± 0.1 and 3.3 ± 0.2 s, respectively). In five slices they originated from the CA1a region and propagated to the CA1b (delay of 1.7 ± 0.2 s) and CA1c (delay of 2.6 ± 0.3 s; Fig. 4b). To assess the synaptic nature underlying their generation, we perfused CA1 mini-slices with high K^+^ aCSF without calcium. Zero Ca^2+^ aCSF reversibly abolished gamma bursts (within 16.1 ± 2 min, *n* = 7 slices from five animals, Fig. 4d), confirming a synaptic mechanism underlying their generation. Furthermore, application of the gap junction blocker carbenoxalone (100 µM) did not significantly alter the gamma bursts (data not shown, *n* = 3). In summary, CA1 gamma bursts appear synaptically mediated but not by glutamatergic transmission, they propagate only locally, and exhibit inherent frequencies (40 Hz) that are higher than typical IED’s.

The above findings point to a synaptic transmission that is rather relying on GABA(A) receptors. We therefore bath perfused the specific GABA(A) receptor antagonist BMI (20 μM) onto CA1 mini-slices with gamma bursts, in the presence of NBQX and D-AP5 to prevent disinhibition induced hyperactivity. This combination strongly suppressed the gamma bursts (see Fig. 5b and Table 2). We next examined whether GABA-induced depolarization as a result of changes in chloride equilibrium potential could underlie the generation of gamma bursts and for that reason perfused slices with modulators of NKCC1, inward co-transporter of Na^+^, K^+^, and Cl^−^ and KCC2, outward co-transporter of K^+^ and Cl^−^. The NKCC1 antagonist bumetanide (10 and 50 μM) significantly reduced all gamma burst parameters (Fig. 5c, d, Table 2), but the KCC2 agonist CLP257 had little effect (Fig. 5e, Table 2). Since it is known that KCC2, under high extracellular K^+^ conditions, may allow influx rather than efflux of Cl^−^ ions^11,17,18^, we instead used the specific KCC2 antagonist VU 0463271 (10 μM). This indeed significantly decreased burst duration, peak amplitude, and gamma power, and also increased the interburst interval, though it also increased somewhat spike frequency (Fig. 5f, see Table 2 for comparison). Taken together, these findings suggest that the gamma bursts are carried by GABA(A) receptor-mediated synaptic transmission that has become excitatory as a result of high extracellular K^+^.

**Table 2:** Pharmacological characterization of gamma bursts in CA1 mini-slices

	Normalized percentage (%)						
	Spikes/burst	Spike frequency	Power of gamma	Peak amplitude	Burst duration	Interburst interval	*n*
BMI (20 μM)	11.3 ± 1.2*	31 ± 3.1*	6.3 ± 0.6*	36.8 ± 1.4*	19 ± 1.8*	71.3 ± 1.2	8 slices from 6 animals
Bumetanide (10 μM)	69.9 ± 2*	93.4 ± 0.9*	59 ± 3.9*	88.9 ± 1.6*	87.1 ± 2.5*	92.1 ± 0.5	8 slices from 6 animals
Bumetanide (50 μM)	58.7 ± 2.4*	31.6 ± 1*	56.8 ± 4.1*	78.5 ± 2.1*	68.3 ± 3.4*	95.8 ± 2.6	11 slices from 8 animals
CLP257(100 μM)	98.5 ± 4.6 (*p* = <0.771)	96.2 ± 2.4 (*p* = <0.180)	105 ± 8 (*p* = <0.741)	92.9 ± 3*	98.2 ± 4.5 (*p* = <0.732)	107.5 ± 1.3*	6 slices from 6 animals
VU 0463271 (10 μM)	96.8 ± 2.5 (*p* = <0.270	109.3 ± 1.4*	84.6 ± 4.6*	93.1 ± 1.6*	89.7 ± 2.3*	121.4 ± 2*	7 slices from 5 animals
Picrotoxin (1 μM)	47.2 ± 1*	119.8 ± 2.2*	85.9 ± 5.6*	86.9 ± 2.4*	35.2 ± 1.3*	68.8 ± 1.7	6 slices from 5 animals
TGOT (0.4 μM)	74.5 ± 1*	97.6 ± 1.3*	46.4 ± 2*	83.5 ± 1*	93.2 ± 3.2*	89.5 ± 0.9	15 slices from 9 animals
TGOT+vasotocin (1 μM)	91 ± 2.2*	97.9 ± 1.7 (*p* = <0.233)	96.6 ± 4.6 (*p* = <0.487)	90 ± 2.2*	95.5 ± 3 (*p* = <0.201)	99.1 ± 0.9 (*p* = <0.445)	6 slices from 5 animals

Table shows the effect of different drugs on the properties of gamma bursts as normalized percentage. Mean ± standard error of the mean; *p = <0.05 significance, paired Student's *t* test.

*BMI* bicuculline methiodide, *TGOT* [Thr4, Gly7] oxytocin.

High extracellular K^+^ may also alter functioning of the neuronal GABA transporter GAT-1 causing it to operate in reverse and, instead of uptake, induce non-vesicular release of GABA^19,20^. The ensuing increase in ambient GABA levels in the extracellular space could enhance tonic conductance through extrasynaptic GABA receptors^21,22^. Tonic activation of such GABA(A) receptors would lead to a steady inflow of Cl^−^ and the resulting increase in [Cl^−^]_I_^23^ could underlie the development of excitatory GABAergic gamma bursting. We studied the involvement of extrasynaptic GABA receptors in gamma burst development by applying picrotoxin at a low concentration (1 μM) at which it is known to only block extrasynaptic GABA receptors^24^. At this concentration, picrotoxin also strongly suppressed gamma bursts and led to a significant reduction in all the parameters studied (Fig. 5g, Table 2). Moreover, to determine if a reversal of GAT-1 transporter played a role in gamma burst generation, the specific GAT-1 blocker SKF89976A hydrochloride (SKF, 40 μM) was applied to CA1 mini-slices showing gamma bursts. It irreversibly abolished all gamma bursts within 50 ± 10.2 min of application (Fig. 5j, Table 2).

Recently, it has been shown that oxytocin, a naturally occurring neuropeptide, can alter [Cl^−^]_i_ accumulation in immature rats by inhibiting NKCC1 during delivery^10^ and by modulating the insertion of KCC2^25^. We therefore also tested oxytocin and found that application of the specific oxytocin agonist TGOT (0.4 µM) significantly reduced all parameters of the gamma bursts (Fig. 5h, Table 2). This effect could be blocked by applying TGOT in the presence of the oxytocin receptor antagonist vasotocin (1 µM, Fig. 5i, Table 2).

## Discussion

Epilepsy and autism exhibit remarkable comorbidity, raising the question whether a common mechanism underlies their development. This might allow shared interventions and enlarge the spectrum of therapies for both diseases. We here report, in a hippocampal slice preparation, an epileptiform activity that consists of bursts with gamma oscillations similar to those previously identified as an underlying cause for behavioral changes in animal models for autism^12^. These bursts are carried by excitatory GABAergic transmission as a result of a change in Cl^−^ equilibrium potential. We show that treatments, previously identified in ASD animal models to target the Cl^−^ equilibrium potential, are also effective against these in vitro epileptiform gamma bursts. Consequently, we found a new potential anti-epileptic role for oxytocin and identified the involvement of tonic signaling through extrasynaptic GABA(A) receptors as a potentially new target for treating ASD.

The epileptiform bursting activity that we found in the CA1 of the rat hippocampal CA1 slice preparation appears to result from a higher concentration in extracellular K^+^ and is blocked by reducing K^+^ to physiological levels (Fig. 4c). Traditionally, high [K^+^]_o_ causes in in vitro slices of the hippocampus the generation of epileptiform events in CA3 that consist of a brief initial sustained phase (ictal tonic) followed by a prolonged intermittent phase (ictal clonic) that lasts tens of seconds^16,26^. Initially these bursts propagate from CA3 to CA1 via SCs, and CA1 subsequently developed distinct population discharges at gamma frequency (gamma bursts), which consistently preceded these events. Similar paroxysms in CA1 have been reported to result from activity-dependent accumulation of [K^+^]_o_^26–28^. Multiple factors such as (i) dense neuronal packing, (ii) exceptionally low extracellular volume fraction, and (iii) CA1’s inability to effectively regulate excess [K^+^]_o_ due to low Na^+^/K^+^ ATPase are thought to contribute toward their generation^28–30^. Our experiments suggest a similar [K^+^]_o_-dependent mechanism that, in contrast to previous studies, depends on synaptic transmission.

In vivo, studies by Bihi et al.^31^ have shown a fast and large increase in CA1 extracellular potassium levels following a 5 Hz stimulus to alveus in urethane-anesthetized animals. Furthermore, in both in vitro and in vivo studies, epileptiform activity is well known to be able to increase the extracellular concentration of potassium from basal levels of 3–5 mM to ceiling levels of 9–12 mM during seizures, recruiting and depolarizing more neurons as the concentration increases^32–34^. Importantly, the scavenging of extracellular potassium is a slow process lasting several seconds which might further lead to prolonged depolarizations^35,36^. In addition, high extracellular potassium leads to swelling of neurons and glial cells resulting in a sudden reduction in extracellular space, which can amplify the extent to which extracellular [K^+^]_o_ increases^37^. In support of this idea, furosemide, a drug known for reducing swelling, is known to suppress seizures (i) in vitro slice models of epilepsy (including electrical kindling in slices, high K^+^, Zero Mg^2+^, Zero Ca^2+^, 4-aminopyridine-induced and bicuculline-induced seizures.), (ii) in vivo seizure models (kainic acid-induced and audiogenic seizure-prone animals), and (iii) in human subjects suffering from intractable seizures (for a review see ref. 38). Taken together, this suggests that changes in extracellular K^+^ can also occur in vivo to play a role in the generation of bursting activity.

Although glutamatergic afferents from CA3 first initiated bursting activity in CA1, anatomical disconnection or pharmacological blockade with glutamate antagonists subsequently promoted rather than suppressed the “gamma bursts” (increasing their frequency from 1/180 to 1/50 s). Similarly, in isolated CA1 “mini-slices,” gamma bursts developed significantly faster than in intact slices (within ~30 instead of >60 min). This suggests a strong suppressive/inhibitory influence from CA3 on the development of CA1 gamma bursts. Computational studies have demonstrated increased [K^+^]_o_ during ictal tonic and decreased [K^+^]_o_ during ictal-clonic periods that are generated in CA3^35^. Their propagation to CA1 may locally decrease [K^+^]_o_ and thus delay the onset of gamma bursts. In addition, CA3 inputs are usually followed by a strong inhibitory drive that can increase the threshold for seizure generation as well^28^. Together, these processes could underlie the inhibition of CA1 gamma bursts by CA3.

An increase in gamma frequencies seems, at first glance, incompatible with the excitation/inhibition theory of ASD as it suggests increased, rather than decreased, levels of inhibition^39^. Changes in Cl^−^ equilibrium potential that lead to excitatory GABAergic transmission could explain this discrepancy, in that they lead both to decreased inhibition and increases in gamma bursts. This can be caused by a high [K^+^]_o_, that activates the Na^+^/K^+^/Cl^−^ co-transporter NKCC1^40^ and/or dysfunction of K^+^/Cl^−^ co-transporter KCC2, thereby allowing intracellular Cl^−^ accumulation^11^. Blocking NKCC1 with bumetanide indeed significantly suppressed the gamma bursts. Though the KCC2 activator CLP257 was not efficient, blocking KCC2 (with bumetanide at higher concentration than used for NKCC1 or with the specific KCC2 blocker VU 0463271) significantly reduced the gamma bursts, suggesting that KCC2 has started working in reverse. In addition, extrasynaptic GABA receptors can contribute to increased [Cl^−^]_i_ as these receptors are highly sensitive to ambient GABA levels^20,41^ and their tonic activation^21,22^ will increase Cl^−^ inflow. Incubation with 1 µM picrotoxin, known to block extrasynaptic but not synaptic GABA transmission^24^, indeed caused a significantly reduction in the amplitude of the gamma bursts. To investigate contributions from tonic GABA release, we tested the involvement of the neuronal GABA transporter GAT-1, which, under high [K^+^]_o_, is known to spill high amounts of GABA into the extrasynaptic space by operating in reverse and producing non-vesicular GABA release^19,20^. Consistently, we found that blocking GAT-1 also reduced gamma burst amplitude. Taken together, these results suggest multiple mechanisms (NKCC1, KCC2, extrasynaptic GABA(A) receptors, and the GAT-1 transporter) through which high [K^+^]_o_ can lead to the accumulation of [Cl^−^]_i_ and the development of gamma bursts.

In our study, application of the selective oxytocin receptor agonist TGOT significantly suppressed the CA1 gamma bursts, while these effects were reduced in the presence of the oxytocin receptor antagonist vasotocin. Recent studies in several rat models for ASD have shown increases in [Cl^−^]_i_ levels and gamma oscillations that can be reverted by oxytocin through its effects on NKCC1 or KCC2^10,12,25^. Furthermore, gamma frequency oscillations in CA1 have been tightly coupled with the firing pattern of parvalbumin expressing fast-spiking interneurons^42^ that are highly sensitive to oxytocin. Oxytocin increases their spontaneous GABA release, thereby depleting GABA available for evoked synaptic transmission^43^. Thus, if activation of fast-spiking interneurons in the CA1 is required to trigger seizure onset as it was shown in the EC^44^, oxytocin could reduce the gamma bursting by preventing sufficient GABAergic transmission. Through both these mechanisms (changes in Cl^−^ equilibrium potential and decreasing evoked GABA release) the generation of CA1 gamma bursts may be efficiently decreased by oxytocin.

The increased prevalence of epilepsy in patients with ASD and the more frequent paroxysmal epileptiform activity in their EEG has led to the belief that ASD and epilepsy may be different clinical manifestations of the same brain pathology. Disturbances in [Cl^−^]_i_ homeostasis have been implicated in ASD^1–5,7,45^ and we here show that the generation of gamma bursts in the CA1 region are likely to originate from similar changes in [Cl^−^]_i_ as a result of extracellular potassium buildup. It is possible that, as in our epileptic model, changes in potassium homeostasis also play a role in ASD. Thus, a number of studies have recently identified dysfunctional K^+^ channels and impaired astrocytic buffering of extracellular potassium in ASD. The resulting increase in extracellular potassium has been proposed a likely trigger for onset of seizures in autism^12,46^. Furthermore, a loss of control of CA1 (e.g., by decreased activity in CA3), in our epileptic model, may also apply in animal models of autism. Thus, recent findings have shown that oxytocin may affect in the CA3 to CA1 projections a change in balance of excitatory vs. inhibitory projections and it is possible that a decrease in its signaling (such as the one on which one of the ASD models is based^12^ may similarly affect the control of the CA3 over the CA1 in these models. In addition, our study shows that drug interventions used against ASD^5,9,10,12^ can also inhibit these epileptiform gamma bursts. These findings raise the question as to how the appearance of epileptiform gamma bursts might affect normal brain function and underlie the appearance of symptoms observed in ASD and thereby the comorbidity with epilepsy.

Gamma frequencies play an important role in the communication and synchronization between different brain regions. The CA1 region is strategically located for communication between CA3 and EC, and directly receives afferents from both regions. Clinically, CA3 is more sensitive to damage than CA1^47^, leading to a loss of control of epileptiform events in CA1^48^. Under such circumstances, it is possible that changes in GABAergic transmission lead to epileptiform discharges enhancing local activity within CA1. This may underlie high levels of autistic traits in humans that have been associated with higher peak gamma frequencies such as better abilities to discriminate differences in line orientations^39^. At the same time, this raises the question whether this enhanced activity remains able to synchronize through the loop between CA1 and EC via the subiculum and the temporoammonic pathway^49,50^. Thus, whereas autism subjects are able to focus on details and recognize these better, they appear less able to integrate these into coherent representations (found in visual, auditory, linguistic, and social cues^51^). It is possible that excitatory gamma bursts reinforce the emphasis on processing that takes place locally, but fail to evoke synchronization with distant regions. Reduced interhemispheric gamma-band coherence has indeed been observed in ASD and is associated with less perceptual integration^52^.

Taken together, we here present an in vitro epileptiform activity in the hippocampal CA1 that can serve as a new preparation for understanding the comorbidity between autism and epilepsy. It suggests an important role for gamma-oscillatory activity as a basis to start disentangling the intricate interactions that underlie cause and effect relationships between the two types of pathologies. From a clinical perspective, our first tests in this preparation reveal a predictive value for treatments of ASD and epilepsy, showing a new potential for oxytocin as an anti-epileptic and suggest that extrasynaptic GABA signaling may be a potential target for the treatment of autism. Thus, we found that the activity in this in vitro model, in addition to providing a new entry point to study comorbidity between autism and epilepsy, can have predictive value in testing new drug candidate treatments for both types of diseases.
